# Identification of *Weissella* species by matrix-assisted laser desorption/ionization time-of-flight mass spectrometry

**DOI:** 10.3389/fmicb.2015.01246

**Published:** 2015-11-05

**Authors:** Meng-Rui Lee, Chia-Jung Tsai, Shih-Hua Teng, Po-Ren Hsueh

**Affiliations:** ^1^Department of Internal Medicine, National Taiwan University Hospital Hsin-Chu Branch, National Taiwan University College of MedicineHsinchu, Taiwan; ^2^Department of Internal Medicine, National Taiwan University Hospital, National Taiwan University College of MedicineTaipei, Taiwan; ^3^Institute of Epidemiology and Preventive Medicine, College of Public Health, National Taiwan UniversityTaipei, Taiwan; ^4^Department of Internal Medicine, Taoyuan General Hospital, Ministry of Health and WelfareTaoyuan, Taiwan; ^5^Department of Graduate Institute of Biomedical Sciences, Chang Gung UniversityTaoyuan, Taiwan; ^6^Department of Laboratory Medicine, National Taiwan University Hospital, National Taiwan University College of MedicineTaipei, Taiwan

**Keywords:** *Weissella confusa*, *Weissella cibaria*, Bacteremia, matrix-assisted laser desorption/ionization time-of-flight mass spectrometry, 16S rRNA

## Abstract

Although some *Weissella* species play beneficial roles in food fermentation and in probiotic products, others such as *Weissella confusa* are emerging Gram-positive pathogens in immunocompromised hosts. *Weissella* species are difficult to identify by conventional biochemical methods and commercial automated systems and are easily misidentified as *Lactobacillus* and *Leuconostoc* species. Matrix-assisted laser desorption/ionization time-of-flight mass spectrometry (MALDI-TOF MS) is increasingly being used for bacterial identification. Little, however, is known about the effectiveness of MALDI-TOF MS in identifying clinical isolates of *Weissella* to the species level. In this study, we evaluated whether the MALDI-TOF MS Bruker Biotyper system could accurately identify a total of 20 *W. confusa* and 2 *W. cibaria* blood isolates that had been confirmed by 16s rRNA sequencing analysis. The MALDI-TOF Biotyper system yielded no reliable identification results based on the current reference spectra for the two species (all score values <1.7). New *W. confusa* spectra were created by randomly selecting 3 *W. confusa* isolates and external validation was performed by testing the remaining 17 *W. confusa* isolates using the new spectra. The new main spectra projection (MSP) yielded reliable score values of >2 for all isolates with the exception of one (score value, 1.963). Our results showed that the MSPs in the current database are not sufficient for correctly identifying *W. confusa* or *W. cibaria*. Further studies including more *Weissella* isolates are warranted to further validate the performance of MALDI-TOF in identifying *Weissella* species.

## Introduction

Members of *Weissella* species are found in various fermented food products and are considered to be potential probiotics (Nam et al., 2002; Fairfax et al., 2014; Kot et al., 2014; Fusco et al., 2015). However, some *Weissella* species, namely *W. confusa*, can cause opportunistic infections in immunocompromised hosts and have been found to be intrinsically resistant to vanomycin, making treatment difficult (Lee et al., 2011). *W. confusa* is most likely an underestimated pathogen because it shares similar staining and biochemical properties with other Gram-positive, catalase-negative bacteria such as *Leuconostoc* and *Lactobacillus* species (Facklam and Elliott, 1995; Shin et al., 2007; Schillinger et al., 2008; Lee et al., 2011; Fairfax et al., 2014).

Traditionally, correct identification of *Weissella* species relies on the implementation of 16S rRNA sequencing (Chelo et al., 2007; Lee et al., 2011). Matrix-assisted laser desorption/ionization time-of-flight mass spectrometry (MALDI-TOF MS) is increasingly being used to identify unusual bacterial pathogens including *Weissella* species isolated from clinical specimens (Lee et al., 2013; Chen et al., 2014; Fairfax et al., 2014; Hsueh et al., 2014). MALDI-TOF MS has the advantage of being faster and more cost-effective than the conventional 16S rRNA sequencing methods and is, therefore, expected to play a more important role in food and clinical laboratories (Steensels et al., 2011). Few studies, however, have addressed and tested the effectiveness of MALDI-TOF MS in identifying *Weissella* species.

In this study, we compared the accuracy of MALDI-TOF MS with that of 16S rRNA sequencing in identifying *Weissella* species.

## Materials and Methods

### Bacterial Isolates

A total of 147 isolates of Gram-positive rods, including *Lactobacillus* and *Leuconostoc* species, that had been recovered from patients with bloodstream infections at the National Taiwan University Hospital (a 2900-bed tertiary care center in northern Taiwan) during the period 2000–2014 were obtained from the hospital’s microbiology laboratory. These isolates had initially been identified as either *Lactobacillus* or *Leuconosto*c species by traditional or commercial automated identification systems as reported previously (Lee et al., 2011, 2015). Among these isolates, 22 isolates were identified as *Weisella* species by colony morphology, a positive Gram stain reaction, growth at 37°C, and negative reactions to pyrrolidonyl arylamidase and leucine aminopeptidase as well as by 16S rRNA sequencing analysis.

### 16S rRNA Gene Sequencing Analysis

Partial sequencing of up to 1475 base pairs of the 16S rRNA gene was performed for species identification of all 147 isolates of *Lactobacillus* and *Leuconostoc* species using the following primers: forward: 5′-AGAGTTTGATCCTGGCTCAG-3′; reverse: 5′-GGTTACCTTGTTACGACTT-3′ (Tsai et al., 2004). The results were compared with published sequences in the GenBank database using the BLASTN algorithm. The closest matches and GenBank accession numbers were obtained. *L. lactis* ATCC 19256 was used as the control strain in each test as described previously (Lee et al., 2011).

### Performance of the MALDI Biotyper

For analysis of the 22 isolates of *Weissella* species by the MALDI-TOF Biotyper (Microflex LT; Bruker Daltonik GmbH, Bremen, Germany), the samples were prepared and analyzed as previously described (Verroken et al., 2010; Hsueh et al., 2014). Briefly, all isolates were incubated in Trypticase soy agar with 5% sheep blood (BAP) (Becton, Dickinson Microbiology Systems, Sparks, MD, USA) and incubated for 48 h at 37°C. Colonies were transferred to a 1.5-ml screw-cap Eppendorf tube containing 300 μl of distilled water and then mixed with 900 μl of ethanol by pipetting. The suspension was pelleted by centrifugation at 13,000 rpm for 2 min, evaporated to dryness, and then reconstituted in 50 μl of 70% formic acid. After incubation for 30 s, 50 μl of acetonitrile (Sigma–Aldrich) was added. The suspension was then centrifuged at 13,000 rpm for 2 min. Next, 1.0 μl of the supernatant was applied to a 96-spot polished steel target plate (Bruker Daltonik GmbH, Bremen, Germany) and dried. A saturated solution of 1.0 μl of MALDI matrix (α-cyano-4-hydroxycinnamic acid [HCCA]; Bruker Daltonik GmbH) was applied to each sample and dried. Measurements were performed with the Bruker microflex LT MALDI-TOF MS system (Bruker Daltonik GmbH) using FlexControl software with Compass Flex Series version 1.3 software and a 60-Hz nitrogen laser (337 nm wavelength). The spectra were collected in the linear positive mode in a mass range covering m/z 1,960–20,132. Spectra ranging from the mass-to-charge ratio (m/z) 2,000–20,000 were analyzed using Bruker Biotyper automation control and the Bruker Biotyper 3.1 software and library (database [DB] 5627 with 5,627 entries). Identification scores of ≥2.000 indicated species-level identification, scores of 1.700–1.999 indicated genus-level identification, and scores of <1.700 indicated no reliable identification. All isolates with discrepant identification results between the molecular and Bruker Biotyper methods were retested twice.

### Cluster Analysis and Creation of New *W. confusa* and *W. cibria* Main Spectra Projection

Clustering analysis of 22 isolates of the two genetically identified *Weissella* species collected from NTUH was performed using ClinProTools 3.0 (Bruker Daltonics GmbH, Bremen, Germany) as previously described (Chen et al., 2014; Hsueh et al., 2014). Because the current MALDI-TOF database failed to make a correct identification of all *W. confusa* and *W. cibaria* isolates, spectra of 3 *W. confusa* isolates were randomly selected from the 20 isolates of *W. confusa* obtained from the NTUH for the creation of main spectra projections (MSPs; database entrance) using Bruker Biotyper MALDI-TOF MS software (Bruker Daltonics) as previously described (Chen et al., 2014; Hsueh et al., 2014). The spectra generated using the 3 isolates were blindly tested against those of the residual 17 *W. confusa* isolates.

## Results

The MALDI-TOF Biotyper system with current reference spectra yielded no reliable identification results (score values ranged from 1.282 to 1.519) among the 20 *W. confusa* and 2 *W. cibria* isolates (**Table 1**). Using the spectra created by the 3 *W. confusa* isolates (Isolates 1, 2, and 3), we further tested the residual 17 *W. confusa* isolates. We found that all of the *W. confusa* isolates with the exception of one (Isolate 17, score value of 1.963) were identified correctly with a score >2 using the three newly created MSPs. The highest scores were achieved with MSP created by *W. confusa* Isolate 1 in the ten *W. confusa* isolates, MSP created by *W. confusa* Isolate 2 in the five *W. confusa* isolates and MSP created by *W. confusa* Isolate 3 in the *W. confusa* isolate.

**Table 1:** Species identification of 22 isolates of *Weissella* species by 16S rRNA sequencing analysis and the MALDI-TOF Biotyper system using preexisting (DB 5627) and newly established databases.

Isolate no.	Original identification results by conventional methods	16S rRNA sequencing		Identification by MALDI Biotyper based on database 5627		Identification by MALDI Biotyper based on newly developed database	
		Results (maximal identity, %)	Accession number	Results	Score value	Results (best match)	Score value
1^∗^	*Lactobacillus* species	*W. confusa* (100)	KF245541.1	NRI	1.361	–	–
2^∗^	*Leuconostoc species*	*W. confusa* (99.8)	EU807756.1	NRI	1.336	–	–
3^∗^	*Leuconostoc* species	*W. confusa* (100)	GU138614.1	NRI	1.298	–	–
4	*Lactobacillus* species	*W. confusa* (100)	KJ476186.1	NRI	1.391	*W. confusa*	2.054
5	*Lactobacillus* species	*W. confusa* (100)	KF245541.1	NRI	1.448	*W. confusa*	2.073
6	*Lactobacillus* species	*W. confusa* (100)	KF245541.1	NRI	1.29	*W. confusa*	2.250
7	*Lactobacillus* species	*W. confusa* (100)	KF245541.1	NRI	1.39	*W. confusa*	2.243
8	*Lactobacillus* species	*W. confusa* (100)	KF245541.1	NRI	1.43	*W. confusa*	2.501
9	*Lactobacillus* species	*W. confusa* (100)	KF245541.1	NRI	1.338	*W. confusa*	2.376
10	*Lactobacillus* species	*W. confusa* (100)	KJ476186.1	NRI	1.503	*W. confusa*	2.113
11	*Leuconostoc* species	*W. confusa* (99.9)	KJ476186.1	NRI	1.303	*W. confusa*	2.395
12	*Leuconostoc* species	*W. confusa* (100)	EU807756.1	NRI	1.35	*W. confusa*	2.101
13	*Leuconostoc* species	*W. confusa* (100)	EU807756.1	NRI	1.362	*W. confusa*	2.04
14	*Leuconostoc* species	*W. confusa* (99.6)	KJ476186.1	NRI	1.391	*W. confusa*	2.332
15	*Lactobacillus species*	*W. confusa* (100)	GU138614.1	NRI	1.416	*W. confusa*	2.333
16	*Leuconostoc* species	*W. confusa* (99.7)	GU138614.1	NRI	1.394	*W. confusa*	2.022
17	*Leuconostoc* species	*W. confusa* (100)	GU138614.1	NRI	1.38	*W. confusa*	1.963
18	*Leuconostoc* species	*W. confusa* (99.8)	KJ476186.1	NRI	1.282	*W. confusa*	2.368
19	*Leuconostoc* species	*W. confusa* (100)	GU138614.1	NRI	1.413	*W. confusa*	2.302
20	*Lactobacillus* species	*W. confusa* (100)	KJ476186.1	NRI	1.382	*W. confusa*	2.355
21	*Leuconostoc* species	*W. cibaria* (100)	AB510747.1	NRI	1.333	–	–
22	*Leuconostoc* species	*W. cibaria* (99.3)	AB510747.1	NRI	1.519	–	–

*^∗^Isolates for creation of *W. confusa* spectra; NRI, no reliable identification.*

**FIGURE 1:**
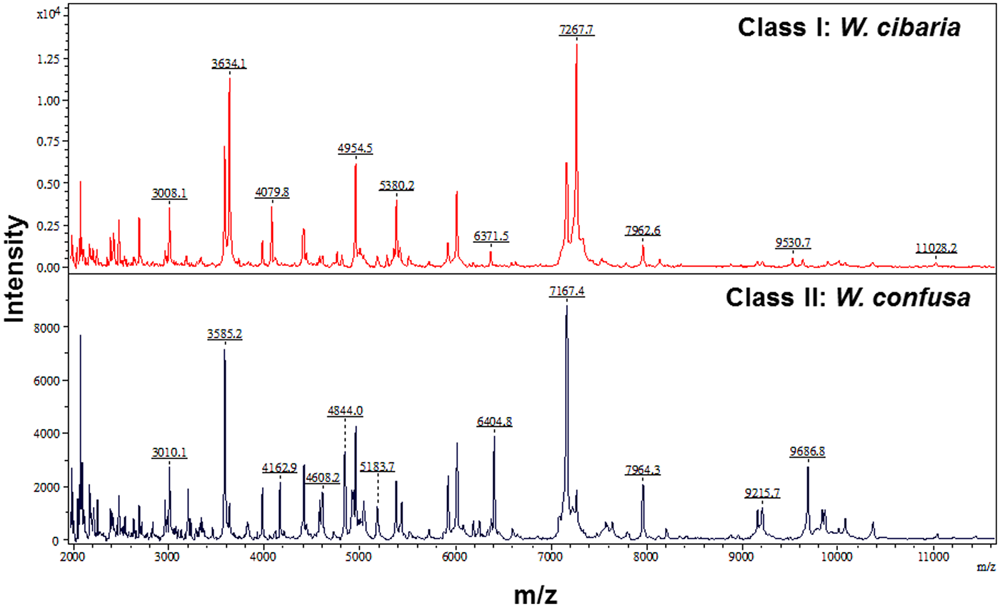
**Two clusters of *Weissella* species spectra, i.e., cluster I (*W. cibaria)* and cluster II (*W. confusa)*, analyzed by clustering analysis of MALDI-TOF MS results.** The absolute intensities of the ions are shown on the *y* axis and the masses (*m/z*) of the ions are shown on the *x* axis. The *m/z* values represent the mass-to-charge ratio.

The characteristic spectra of *W. confusa* (Isolate 8) and *W. cibria* (Isolate 22) are illustrated in **Figure 1**. The characteristic peaks for *W. cibria* were 3008.1, 3634.1, 4079.8, 4954.5, 5380.2, 6371.5, 7267.7, 7962.6, 9530.7, and 11028.2. The characteristic peaks for *W. confusa* were 3010.1, 3585.2, 4162.9, 4608.2, 4844.0, 5183.7, 6404.8, 7167.4, 7964.3, 9215.7, and 9686.8.

## Discussion

We found that current reference spectra in the MALDI-TOF database were much less accurate than 16s rRNA sequencing in correctly identifying *W. confusa* and *W. cibaria*. Using the *W. confusa* MSPs created by our database, we were able to correctly identify all *W. confusa* isolates.

It is notoriously difficult to identify *W. confusa*. The 16S rRNA sequencing method is considered the standard identification method, although other methods have shown to be able to accurately identify *W. confusa*. A novel PCR-based identification method using amplified fragment length polymorphism (AFLP)-derived marker was shown to have 100% specificity in identifying *W. confusa* (Fusco et al., 2011). MALDI-TOF has also been shown to correctly identify the organism in blood samples (Lee et al., 2013; Fairfax et al., 2014). Despite satisfying results, it has to be kept in mind that the tested case numbers were small and only five *Weissella* species are listed in the MALDI-TOF database, namely *W. confusa, W. halotolerans, W. kandleri, W. minor*, and *W. viridescens*. Stevenson et al. (2010) reported that only one *W. confusa* isolate achieved a score value of 1.7, indicating an identification only at genus level. In our study, all of the *W. confusa* isolates that had been correctly identified by 16S rRNA sequencing failed to be identified with MALDI-TOF using the current database. The discrepancy between the two identification methods remains to be solved. Further studies comparing the results between 16S rRNA sequencing and MALDI-TOF are warranted.

The most well-studied food product in association with *Weissella* species is kimchi, a traditional Korean food. Lactic acid bacteria, belonging to the genera *Leuconostoc, Lactobacillus*, and *Weissella*, are considered to be the most likely organisms responsible for kimchi fermentation (Lee et al., 2005; Jung et al., 2013; Kwak et al., 2014), and studies have shown that *W. confusa* is involved in the early and middle stages of kimchi fermentation (Lee et al., 2005; Kim et al., 2012). *W. confusa* has also been detected in food products from other countries, such as fermented milk products in Ghana, sourdough products in Italy, fermented soy sauce in Taiwan, and in soy sauce brine in Malaysia (Corsetti et al., 2001; Akabanda et al., 2013; Wei et al., 2013; Sulaiman et al., 2014).

Although *W. ceti* is well known in veterinary medicine for causing weissellosis in rainbow trout (Snyder et al., 2015), to the best of our knowledge, only *W. confusa* has been linked to human invasive infections. Therefore, other *Weissella* species are probably safer alternatives to *W. confusa* as probiotics.

Bacteremia due to *W. confusa* is rare and tends to occur in severely immunocompromised hosts, such as patients who have received organ transplants (Harlan et al., 2011; Lee et al., 2011, 2013; Salimnia et al., 2011; Fairfax et al., 2014). Immunocompromised hosts undergoing abdominal surgery have also been shown to be at particular risk since abdominal surgery results in the breakdown of intestinal mucosa (Lee et al., 2011). Given the findings of our study, the incidence of bacteremia due to *W. confusa* is underestimated because of the difficulties in making a correct identification with commercial and biochemical methods. The findings also suggest that immunocompromised patients should refrain from consuming fermented foods.

In our previous study, the results of 16S rRNA sequencing analysis showed that 10 of 43 blood isolates of catalase-negative, vancomycin-resistant coccobacilli were actually *W. confusa* (Lee et al., 2011). In this study, all *Weissella* species were either identified as *Lactobacillus* or *Leuconostoc* species by commercial automatic systems or traditional biochemical methods. This finding is in concordance with a phylogenetical study by Chelo et al. (2007), who showed that *Leuconostoc* species and *Lactobacillus* species were close to *W. confusa* in neighbor-joining tree by 16S rRNA sequencing. The applications of 16S rRNA sequencing may lead to a better understanding of the incidence of bacteremia due to *Weissella* species.

During the study period, the actual number of bacteremia due to *Weissella* species might be under-estimated. In this study, we did not perform 16S rRNA sequencing on bacteremic isolates of Gram-positive cocci, including viridians group streptococci, *Pediococcus*, and *Gemella* species. Some *Weissella* species were misidentified as these organisms by conventional identification methods (Ruoff, 2002; Lee et al., 2011). In this study, 16S rRNA sequencing was only performed on bacteremic isolates of *Lactobacillus* and *Leuconostoc* species, which were the most closely relevant species and thus were considered the most possible bacteria *W. confusa* could be misidentified as (Chelo et al., 2007; Lee et al., 2011).

## Conclusion

The MALDI Biotyper system is less accurate that 16S rRNA sequencing in correctly identifying *Weissella* species. *W. confusa* is an underestimated cause of bacteremia due to its tendency to be misidentified. More extensive epidemiologic studies with molecular methods are needed to reveal the true prevalence and incidence of invasive *W. confusa* infections. Also, studies involving more clinical *Weissella* isolates are needed to explore the effectiveness of the MALDI Biotyper system in identification of *Weissella* species.

## Conflict of Interest Statement

The authors declare that the research was conducted in the absence of any commercial or financial relationships that could be construed as a potential conflict of interest.
